# Milk metabolite composition of a semi-captive population of Asian elephants

**DOI:** 10.1098/rsos.240930

**Published:** 2024-10-30

**Authors:** Laura Galante, Diogo João Franco dos Santos, Elisabeth Mikkonen, Jeannie Horak, Zorica Stijepic, Hans Demmelmair, Andrea Vielhauer, Berthold Koletzko, Htet Thi Zaw, Win Htut, Virpi Lummaa, Mirkka Lahdenperä

**Affiliations:** ^1^ School of Medicine, Swansea University, Swansea SA2 8PP, UK; ^2^ Department of Biology, University of Turku, Turku FI-20014, Finland; ^3^ Department of Metabolic and Nutritional Medicine, Ludwig-Maximilians-University Munich, Medical Center, Munich D-80337, Germany; ^4^ Myanma Timber Enterprise, Yangon 11011, Myanmar

**Keywords:** *Elephas maximus*, milk composition, milk metabolome, lactation physiology, animal nutrition

## Abstract

Lack of maternal milk commonly leads to Asian elephant calves’ death in captivity. Currently, available supplements seem inefficient. Hence, we aimed at characterizing the composition of Asian elephant milk to provide information on calves’ nutritional needs. Seventy milk samples from 22 Asian elephants living in semi-captivity in their natural environment in Myanmar were collected. Samples were analysed through various techniques including liquid chromatography tandem mass spectrometry, gas chromatography-flame ionization detector, and bicinchoninic acid assay to determine total protein content and various metabolites. Associations with lactation stage (months postpartum) were investigated through repeated measure mixed models. We identified 160 compounds: 22 amino acids, 12 organic acids of the tricarboxylic acid cycle, 27 fatty acids, 15 acyl-carnitines and 84 phospholipids. The milk contained substantial amounts of free glutamate (median: 1727.9, interquartile range (IQR): 1278.4 µmol l^−1^) and free glycine (2541.7, IQR: 1704.1 µmol l^−1^). The fatty acid profile was mostly constituted by saturated fatty acids, particularly capric acid (40.1, IQR: 67.3 g l^−1^). Milk samples also contained high amounts of carnitines, phospholipids and organic acids. The wide array of metabolites identified and quantified, some of which present high concentrations in the milk from this species as opposed to other species, suggests underpinning physiological functions that might be crucial for the survival of Asian elephant calves.

## 1. Introduction

Milk and the lactation process have evolved to support the survival and development of offspring during their most vulnerable period [1], by meeting the physiological requirements specific to each mammalian species [2]. This is evident when considering comparative milk compositional studies showing that milk significantly differs across species, not only in terms of energy content but also in terms of concentration and structure of specific bioactives [3–6]. Such differences are likely to have important functions in the growth of the offspring and are driven by differences in ecology, development and physiology across different species [7].

For example, the development of large body size and brain most likely requires an energy-dense milk and some specific set of other nutritional and non-nutritional components [8–11]. Elephants are the largest terrestrial mammals and together with humans and great apes display a large and very complex brain [12], which has led to the evolution of very long lactation that lasts between 2 and 8 years in the wild, usually continuing until the mother gives birth to a new calf [13,14]. Behavioural and physiological aspects of lactation, including the extent of milk yield produced by elephant cows, have not been overly studied, however, previous literature on African elephants suggested that milk yield/demand might differ for male vs female calves [13]. Some lactation physiology for these animals has been previously collated by Osthoff in a chapter of the book entitled ‘*Elephants: ecology, behavior and conservation*’ [15]. Briefly, the author reports on milk compositional variations from studies on both African and Asian elephants and according to these changes, particularly in relation to fat content, identifies three lactation stages. Similarly, three lactation stages have been identified by the same author and others in a different publication (colostrum, early to mid-lactation and late lactation) [16], with the middle stage (up to 12 months) where most changes in the nutritional content of the milk happen. However, while Osthoff reports no impact of maternal traits on milk composition, we previously found that size, age, birth origin and parity were significantly associated with the composition of the milk in Asian elephants [17]. As such, it is important that analysis of milk compositional variation across lactation takes into account maternal and offspring factors that might modify the outcome. Such variability in results related to the effect of lactation time on milk composition warrants deeper investigations. Additionally, while research on macronutrients in elephant milk has yielded an overall understanding of the general composition of the milk (extensively reported elsewhere [15]), recent research particularly on human milk, is shedding light on the importance of milk bioactive components in relation to growth, development, and developmental origin of health and diseases [18]. For instance, research on both animal models and humans has shown that the presence of milk fat globule membrane in maternal milk [19] has important implications for infant neurodevelopment [9,20,21]. Similarly, taurine, of which maternal milk is the only source for the nursing offspring, also seems to promote neurodevelopment and improved cognition according to both animal and human studies [22–24]. At the same time, research on primates has shown that the exposure to components in maternal milk across lactation can have different effects on neurodevelopmental outcomes in relation to the maternal environment and the offspring characteristics [25]. Taken together, this research has highlighted the importance of considering milk composition as an important factor for the developmental trajectories (including the development of health and disease) of the offspring. This phenomenon is known as lactocrine programming [26,27]. The accumulation of evidence on this topic is applicable to both human health and animal welfare, particularly when lack of maternal milk is among the primary causes of offspring mortality.

In the case of elephants, hand-rearing can be difficult and often unsuccessful [15,28]. High calf mortality in captivity owing to lack of maternal milk [24] and difficulties in hand rearing of elephants have previously been observed [25–28] highlighting the need for better understanding of the lactation process and milk composition for these animals. Previous reports on calves fed with milk replacers suggest that calves have been subject to bone fracture, diarrhoea and infections, all conditions that can be associated with nutritional deficits. Although previous research shows that the majority of maternal-to-offspring transfer of immunity is transplacental [29], recent literature on the hand-rearing of this species reports that colostrum is crucial for the survival of calves and that denial of any milk provision from mother to calf within 24 h post-birth can result in compromised immunity [30]. Moreover, an analysis of currently available milk replacers by Ward *et al.* [31] suggested that these formulations present a large variability in terms of macronutrient percentage (i.e. fat, lactose and protein). However, such comparison with elephant milk was done using two cows and three lactations spanning from birth to six months, which is a rather limited amount of time in the context of elephant lactation. More recently a hand-raising guide for orphaned Asian elephant has been published online, with a chapter written by Dierenfeld, entirely dedicated to feeding [32]. Here an extensive comparison is made between different milk replacers that are commonly used to hand-rear orphaned calves. Such analysis confirms what was stated by Ward *et al*. showing a large variation in both macronutrients and some vitamins across the formulations used. Most importantly, recent developments in metabolomic technologies are starting to fill the gap around the compositional differences of early life nutritional products for humans showing potential pathways in which different types of feed might affect physiology [33]. As the Asian elephant is currently considered an endangered species with less than 50 000 wild individuals remaining according to the World Wide Fund for nature [34] and more than 25% of the total population living today in captivity [35], defining the early life requirements of the species in order to improve welfare of captive animals can help conservation efforts. For instance, Myanmar hosts the largest semi-captive Asian elephant population [36], most of which is owned by the Myanma Timber Enterprise (MTE). MTE semi-captive elephants live in forest camps, where they are used for logging work during the day, while at night they forage unsupervised with their family groups in the forest. While mortality rates in this population are lower than those in zoo animals [37], young individuals remain at high risk [38]. Maternal death [39] and lack of maternal milk are known to significantly decrease calf survival [38], making access to maternal milk a substantial problem for managing semi-captive and captive elephant populations [5,31,38–40]. Particularly, we previously found that mortality is highest during the first and second year of life (with one in four calves dying during the first year owing to maternal agalactia and/or general weakness of the newborn) [38]. This corresponds to the time when calves were most dependent on maternal milk and maternal care [32].

Yet to date, only a few studies have analysed milk from Asian elephants. While these studies have highlighted the complexity and uniqueness of Asian elephant milk composition by showing its differences in terms of oligosaccharides [41–43] and other compounds [28] in comparison to other mammals’ milk, its compositional profile is still largely unknown and the analysed lactation span is limited in terms of length and animals used. Moreover, current data is available mostly on isolated nutrient profiles without an in-depth analysis of the associated metabolome. While similarly to humans, the composition of milk is likely to change between mothers and within mothers [44], and across offspring [17], the existing studies have been carried out in very small sample sizes (*n*<10 elephants) typically from zoo environments. In these circumstances, the elephants are not consuming their natural diet and are more likely to suffer from abnormal behaviour and health issues [45], which ultimately can reduce their natural lifespan [37]. Diet, health and environmental factors have all been shown to be associated with milk composition in humans and animal models [46–50]. Therefore, the difference in diet and lifestyle between wild and captive elephant populations is also likely to influence the composition of the milk and therefore to obstruct the full characterization of a ‘normal’ compositional profile for this species. This knowledge gap is therefore reflected on artificial formula milk already existing on the market which being formulated on limited and ‘patchy data’ possibly fail to provide important compounds currently understudied.

Therefore, the aim of this study was to use a multi-omics approach (metabolomics and lipidomics) in order to provide a comprehensive nutritional characterization of milk metabolites [51] from 22 Asian elephant cows living in semi-captive conditions in Myanmar, where they are used to carry out work within the local logging industry, but forage, breed and socialise unsupervised in their natural habitat. These metabolites include fatty acids, amino acids and organic acids. These elephants provided repeated milk samples at different lactation stages (from 1 to 55 months after birth) offering the opportunity to analyse the changes in milk metabolite composition which might occur across the lactation period. The studies that to date analysed the changes of elephant (both African and Asian) milk composition across lactation suggest that protein and lipid content of milk increases across the lactation period, which slightly varies across the different studies [16,17,44,52,53]. We therefore expected to see similar trends in the present population. Most importantly with the present study, we expected to shed light on previously unidentified and unquantified metabolites that might be found in high concentrations in milk from Asian elephants, therefore indicating a possible physiological function in the growth and development of Asian elephant calves and/or of lactating mothers.

## 2. Material and methods

### 2.1. Study design

The present study followed an observational design to explore the milk composition of semi-captive Asian elephants (*Elephas maximus*) from Myanmar.

### 2.2. Elephant population

The study included 22 Asian elephant females employed as logging elephants by MTE in Kawlin, Myanmar. MTE elephants are used during the day as riding, transport, and draught animals following strict working regulations [54] but at night and during rest periods, they forage in the forest unsupervised. The females included in the current study were not working at the time of the study. The breeding rates for the population are natural and not managed by humans, and many captive-born calves are thought to be sired by wild bulls. Calves born in captivity are cared for by their biological and allo-mothers [55,56]. Mothers and calves are never separated and the calves are allowed to suckle on demand until natural weaning occurs or until taming, which happens at 4 years of age [35]. Myanmar is characterized by three seasons: the cool and dry season (from November to February), the hot and dry season (from March to May) and the rainy monsoon season (from June to October). Mothers rely primarily on natural foraging, with limited additional dietary supplementation. The nutrient composition of the primary forage available to mothers has been described before [17].

Table 1 shows the main characteristics for each mother in the study. Maternal age ranged from 13 to 58 years with an average of 34 (±13). Mothers’ parity at first milk collection varied, with a maximum of nine previous births. However, most mothers (73%) had three or less previous calves and nine mothers (40%) had their first calf. Four mothers (18%) were pregnant during the study time and two of them gave birth before the end of the milk collection period. Calves were almost equally distributed between males (46%) and females (54%). Finally, the lactation period at milk collection varied from 1 to 55 months postpartum, with an average lactation time of 21 (±14) months postpartum at first sample collection (May 2020). The two mothers who gave birth during the study period provided at least two very early lactation samples each.

**Table 1.** Mothers, calves and milk collection details (species: *Elephas maximus*). (M, male calf; F, female calf.)

mother ID	mother date of birth	calf date of birth	parity at first milk collection	calf sex	*n* of samples	date of first collection	lactation stage at first collection (months)	lactation stage at last collection (months)
1	01/01/1963	13/07/2019	7	M	4	01/05/2020	10	14
2^a^	01/01/1966	04/08/2020	9	F	4	01/05/2020	51	55
3	21/03/1969	03/04/2019	7	M	2	01/06/2020	14	15
4	01/01/1966	25/01/2017	5	M	4	01/05/2020	40	44
5	11/02/1976	08/09/2018	2	M	4	01/05/2020	20	24
6^b^	12/05/1977	03/06/2020	3	F	4	01/05/2020	39	2
7	02/01/1978	05/08/2019	3	M	4	01/05/2020	9	13
8	04/04/1977	28/09/2017	6	F	4	01/05/2020	32	35
9	17/08/1987	25/02/2018	3	M	2	01/05/2020	27	28
10	03/10/1989	01/11/2019	4	F	4	01/05/2020	6	10
11	24/08/1989	01/04/2019	2	F	1	01/06/2020	14	14
12	10/09/1990	03/10/2019	3	M	4	01/05/2020	7	10
13	16/08/1990	08/08/2018	1	F	2	01/05/2020	21	22
14	09/03/1991	27/08/2018	3	F	4	01/05/2020	20	24
15	10/10/1991	25/10/2019	1	M	1	01/07/2020	8	8
16^a^	01/04/1993	31/05/2021	1	M	2	01/07/2020	46	48
17^b^	04/06/1993	11/06/2020	1	F	4	01/05/2020	41	2
18	11/03/1997	17/09/2018	1	F	2	01/05/2020	20	21
19	01/04/1998	08/05/2019	1	F	4	01/05/2020	12	15
20	26/06/1998	30/05/2019	1	M	4	01/05/2020	11	14
21	01/06/2000	06/02/2019	1	M	2	01/07/2020	17	18
22	10/07/2006	07/10/2019	1	F	4	01/05/2020	7	10

^a^ Pregnant during the study,

^b^ Gave birth to a new calf during the study.

### 2.3. Milk sample collection

Milk samples were collected on the same day once per month (*n* = 70 in total) in May–June 2020 (hot season) and July–August 2020 (rainy monsoon season). All milk samples were collected at or after six months postpartum during the first two months of collection. In order to collect the sample, the calf was allowed to suckle for a few minutes to stimulate milk let-down, the calf was then momentarily displaced in order to allow the collection of around 20 ml of milk via manual expression in a plastic container. Collected samples were moved to 15 ml falcon tubes and transported on ice within 24 h of collection. All vials were stored at −20°C and kept frozen until they were shipped on dry ice to Finland in October 2020. In Finland, samples were thoroughly mixed and aliquotted in 0.5 and 1 ml test tubes and stored at −80°C until analysis or until transfer to the University of Munich Medical Center, where they underwent metabolomic profiling.

### 2.4. Milk analysis

#### 2.4.1. Total protein quantification

Total protein concentration was analysed through the bicinchoninic acid assay (BCA) method (Pierce BCA Protein assay kit, Thermofisher) after validation for use in elephant milk samples. Validation experiments were conducted on both skimmed and whole milk, and different dilution factors (between 1 : 20 and 1 : 50). Whole milk, diluted 1 : 30 was identified as the optimal preparation for total protein detection with this method, yielding recovery rates between 97 and 110% and intraplate quality control (QC) coefficient of variation (CV) <1%. Validation was conducted in triplicate on milk samples from three different elephant mothers and elephant serum was used as QC. After validation, sample analysis was conducted in single (intraplate QC CV<10%) and was run according to the manufacturer instructions using 10 µl of milk per assay.

#### 2.4.2. Multi-platform liquid chromatography tandem mass spectrometry

All solvents were of liquid chromatography tandem mass spectrometry (LC-MS) grade and purchased from Sigma-Aldrich (Schnelldorf, Germany). In total, 200 µl polymerase chain reaction (PCR) 96-well microplates (conical, skirted) were from Axygen (San Francisco, USA) and 1.2 ml 96 deep-well plates (round bottom, low profile) were from BRAND (Wertheim, Germany). Polypropylene PCR microplate foil was from RatioLab (Dreieichen, Germany). PCR microplate aluminum heat sealing foil and the HeatSealer S100 were from Eppendorf (Hamburg, Germany). For elephant milk analysis, 50 µl of elephant milk was directly pipetted into 450 µl of a methanolic internal standard mixture in a 1.3 ml 96 deep well plate. Flocculent protein precipitation was achieved by draw and eject with the pipette tip (20×). After shaking of the well plate at 700 rpm at 25°C (ThermoMixer C with well plate adapter from Eppendorf) and storage at 4°C for 20 min, the well plate was centrifuged for 5 min at 3000 rpm at 22°C (centrifuge ROTINA 380R from Andreas Hettich GmbH & Co. KG (Tuttlingen, Germany)) and the supernatant was transferred to a new 1.3 ml 96 deep well plate. The protein pellet was re-suspended with 50 µl water, followed by addition of 75% methyl-tert-butyl ether in methanol. Again, protein precipitation was achieved by draw and eject with the pipette tip (20×). All other steps were performed as described above. The polar metabolite and lipid extracts were pooled and stored at −30°C prior to high performance liquid chromatography-electrospray ionization tandem mass spectrometry (HPLC-ESI-MS/MS) and flow injection analysis-electrospray ionization tandem mass spectrometry (FIA-ESI-MS/MS) analysis.

For analysis, samples were prepared in 200 µl micro-well plates using aluminium heat sealing foils. Per well plate analysis (batch), four control plasma (CPI and CPII) and six QCs (elephant milk sample pools) were used for system performance check and for statistical data cleaning, respectively. Besides that, 74 samples, 10 calibrant solutions, one system blank (water) and one internal standard blank were included per analysis batch. Amino acid HPLC-ESI-MS/MS analysis was performed according to Newton *et al.* [57] with increased highest calibrant concentration of 5 mmol l^−1^ for glycine and glutamic acid, compared to 1 mmol l^−1^ for all other amino acids. HPLC-ESI-MS/MS analysis was done on an Agilent 1100 HPLC system which comprised of a binary pump (G1312A), a degaser (G1379A), column oven (G1316A) and a temperature controlled well plate autosampler (G1367A with G1330B) in combination with an API 2000 triple quadrupole mass spectrometer from Sciex (Ontario, Canada). Organic acid [58], acylcarnitine [59] and phosphor-cholines (PCaa, PCae, LPCa, LPCe and SM; a = acyl and e = ether) [60] were performed on an Agilent 1260 HPLC system comprising of a binary pump (G1312B), degaser (G1379B) and multisampler (G7167A) from Agilent (Waldbronn, Germany) combined with a MayLab MistraSwitch column oven from MayLab (Vienna, Austria) and a QTRAP 4000 mass spectrometer with a Turbo V ESI source from Sciex (Ontario, Canada). Note that the phosphor-cholines were analysed with an FIA method.

MS-data acquisition was performed with Analyst 1.6.3 and MS-data quantification with MultiQuant 3.0.3 from Sciex. For peak integration, isotope correction and quantification of FIA-ESI-MS/MS generated phospho-choline lipid data an in-house developed R-script was used. R and R-Studio statistical scripting language, version 4.1.0 and version 2023.06.0.421 (CRAN) were used [61].

#### 2.4.3. Gas chromatography-flame ionization detector analysis

Total milk fatty acids were analysed as previously described [62]. Briefly, 50 µl of milk were combined with methanolic HCl and internal standard (triundecanoin) containing hexane. Esterified and free fatty acids were transferred into their methyl esters by heating to 100°C. After a cooling phase separation was achieved by the addition of water and hexane. The fatty acid methyl-ester containing hexane phase was used for gas chromatography (Agilent 7890 GC; BPX−70 column). This enabled quantification of fatty acids (g l^−1^) with chain lengths from 8 to 22 carbon atoms. Fatty acids (CX:Yn-Z) are described by the total number of their C-atoms (X), the number of double bonds (Y) and the number of carbon atoms between the methyl end and the last double bond (Z).

### 2.5. Statistical analysis of data

Summary values of each component are expressed as median (interquartile range, IQR), as the concentrations of most compounds in the milk are not normally distributed. Therefore, all compounds were log-transformed (log_10_) in order to carry out statistical analyses. Unadjusted linear mixed model repeated measure analysis was applied to examine the associations between the most abundant milk components and the lactation stage (months postpartum) across the collection time-points. In addition, multivariate analysis (MANOVA) was carried out on individual packages of metabolites (i.e. amino-acids, fatty acids, carnitines, phospholipids, organic acids of the tricarboxylic acid (TCA) cycle). In this case as it was not practical to fit a repeated measure model owing to the size of the dataset, the median of the concentration of each metabolite across the collected samples for each mother was calculated, together with the median lactation time (months postpartum). For this purpose, models were fitted with and without the two mothers who gave birth on the study (elephant IDs 6 and 17). These mothers transitioned from late lactation to early lactation. When including these mothers in the model we only accounted for the early lactation samples as these were the only mothers providing samples during the first two months postpartum, making these samples more valuable than the late lactation samples. The median of the outcome variables was log-transformed in order to fit the models. Unadjusted models were first tested with only lactation time as a covariate. We then adjusted the model for parity and sex of the calf as they were previously found to be associated with milk composition [17]. Statistical significance was set at *p* ≤ 0.05. All statistical analyses were performed using IBM SPSS (version 29). Graphs were created with a SGPLOT procedure in SAS (SAS Institute Inc., 9.4, 2014) and ggplot procedure in R-Studio (version 4.1.1). Heatmaps were created with Excel (Microsoft 365).

## 3. Results

### 3.1. Overall composition

**Figure 1.**
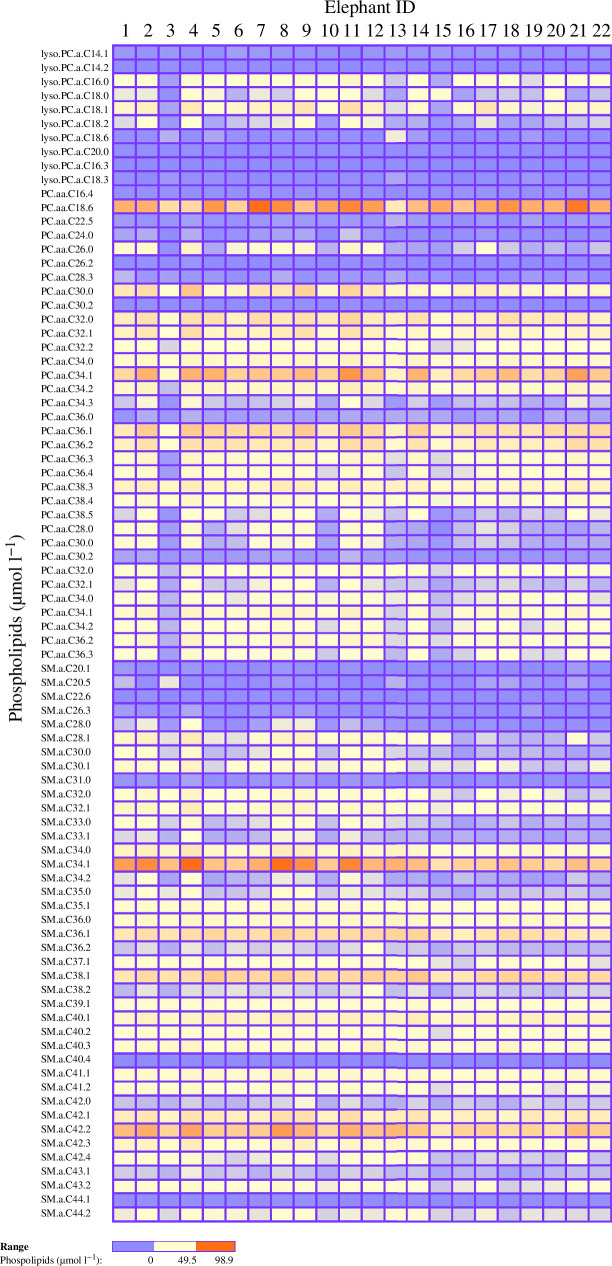
Between-individuals variation of milk amino acids, fatty acids, acylcarnitines and TCA cycle compounds in the 22 Asian elephant mothers included in the study. Columns represent elephants while rows represent metabolites. The red colour signals the highest concentrations of metabolites while blue colour signals the lowest concentrations of metabolites. Values represent average metabolite concentrations across all collections for each elephant (*Elephas maximus*).

**Figure 2.**
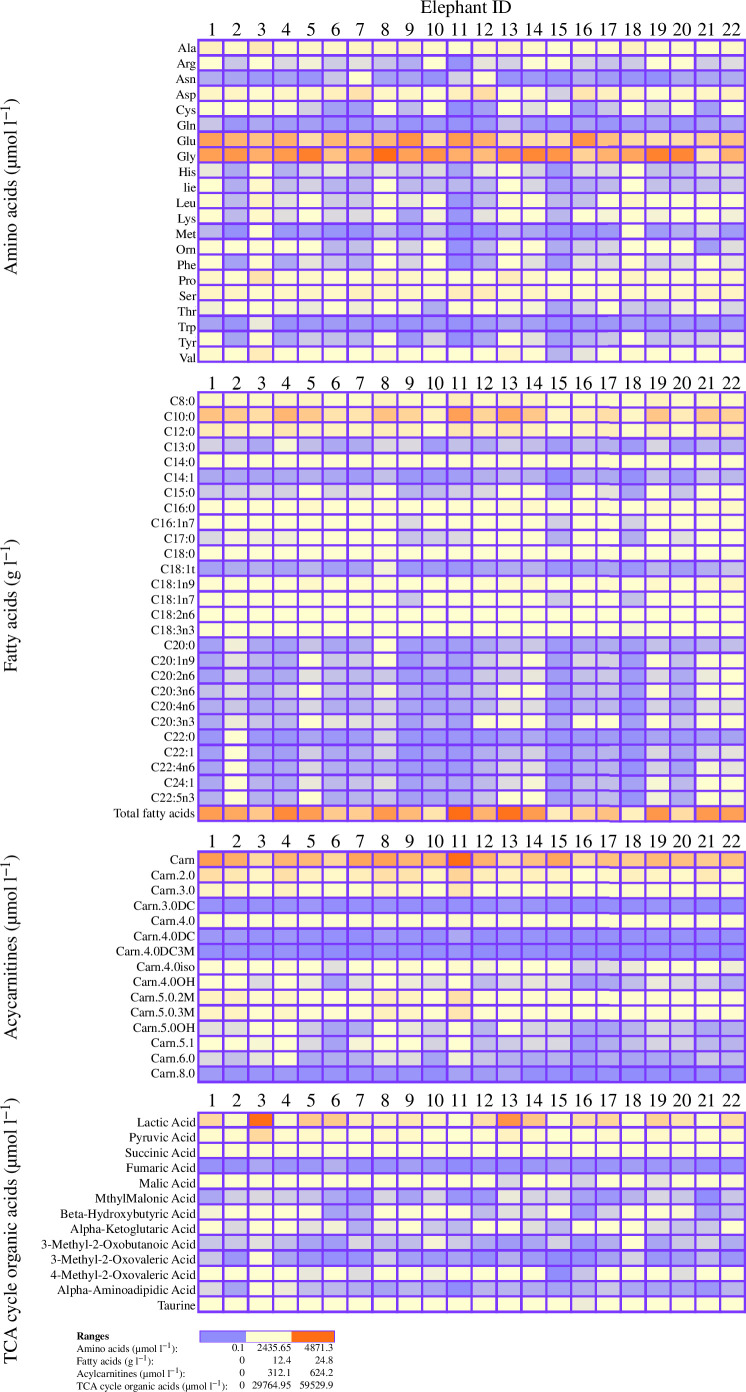
Between-individuals variation of milk phospholipids in the 22 Asian elephant mothers included in the study. Columns represent elephants while rows represent metabolites. The red colour signals the highest concentrations of metabolites while blue colour signals the lowest concentrations of metabolites. Values represent average metabolite concentrations across all collections for each elephant (*Elephas maximus*).

Table 2 summarizes the composition of the analysed milk samples across all animals and gives a general idea of the Asian elephant milk metabolome. The median concentration of protein was 3.8 (1.4) g 100 ml^−1^ and the concentration of total fatty acids was 72.4 (115) g l^−1^. Notably some metabolites displayed higher concentrations than others. All the identified amino acids displayed relatively high concentrations (>3 µmol l^−1^) but glutamate (1727.9 (1278.4) µmol l^−1^) and glycine (2541.7 (1704.1) µmol l^−1^) were particularly high. Other abundant amino acids included alanine (220 (227.2) µmol l^−1^), aspartic acid (284.2 (502.1) µmol l^−1^), proline (161.9 (227.6) µmol l^−1^) and serine (201.5 (263.4) µmol l^−1^). The majority of the fat content was constituted by saturated fatty acids, as shown in table 3, particularly capric acid (40.1 (67.3) g l^−1^) and lauric acid (13.3 (21) g l^−1^). Oleic acid was the most abundant unsaturated fatty acid (2.8 (5.0) g l^−1^). Carnitine displayed a median concentration of 287(188.6) µmol l^−1^, while acetyl-carnitine (Carn.2) was the next most abundant acylcarnitine with concentration of 43(83.5) µmol l^−1^. Among the phospholipid metabolites, phosphatidylcholine (PC).aa.C18.6 was the highest (28.5 (32.6) µmol l^−1^), followed by sphingomyelin (SM).a.C34.1 (25 (28.5) µmol l^−1^), SM.a.C42.2 (21.1 (18.9) µmol l^−1^), SM.a.C38.1 (15.7 (9.3) µmol l^−1^) and SM.a.C36.1 (12.7 (7.7) µmol l^−1^). Finally, in the context of glycolysis and TCA cycle, the compounds with the highest concentrations were lactic acid (2511 (24 596) µmol l^−1^), pyruvic acid (745.8 (3066.6) µmol l^−1^), succinic acid (187.6 (454.2) µmol l^−1^) and taurine (55.3 (60.3) µmol l^−1^). Figures 1 and 2 show between-individual variations of the milk metabolite composition in our sample.

**Table 2.** Summary of the concentration of the components found in the elephant milk samples. (*n* = 70 milk samples (May–August 2020) from 22 females of *Elephas maximus*. C, carbon; PC, phosphatidylcholine; SM, sphingomyeline; a, acyl; e, ether)

compound	median (IQR)	min-max
protein and amino acids		
total protein (g 100 ml^−1^)	3.8 (1.4)	1.8–6.4
alanine (µmol l^−1^)	220 (227.2)	54.5–1011.2
arginine (µmol l^−1^)	32.5 (35.2)	2.6–133.5
asparagine (µmol l^−1^)	5.4 (15.7)	0.5-338.2
aspartic acid (µmol l^−1^)	284.2 (502.1)	8.5–1556.44
cysteine (µmol l^−1^)	15.6 (45.9)	0.7–224.2
glutamine (µmol l^−1^)	5.4 (4.9)	1.7–69.1
glutamate (µmol l^−1^)	1727.9 (1278.4)	34.1–3122.2
glycine (µmol l^−1^)	2541.7 (1704.1)	553.4–4871.3
histidine (µmol l^−1^)	30 (26.3)	3.9–160.2
isoleucine (µmol l^−1^)	17.7 (19.7)	10–310
leucine (µmol l^−1^)	33.4 (83.6)	3.2–597
lysine (µmol l^−1^)	38 (59.5)	3.3–284.9
methionine (µmol l^−1^)	4.3 (10.1)	0.1–110.8
ornithine (µmol l^−1^)	30.7 (67.3)	1.1–241.8
phenylalanine (µmol l^−1^)	19.4 (46.2)	0.2–267.1
proline (µmol l^−1^)	161.9 (227.6)	39.3–925.01
serine (µmol l^−1^)	201.5 (263.4)	81.8–758.9
threonine (µmol l^−1^)	39 (52)	7.7–144.5
tryptophan (µmol l^−1^)	5.4 (4.1)	2.7–69.6
tyrosine (µmol l^−1^)	17.4 (30.9)	1.7–228.1
valine (µmol l^−1^)	59.5 (82.1)	15.7–751.8
fatty acids		
total fatty acid (g l^−1^)	72.4 (115)	7.2–293.8
C8:0 (g l^−1^)—caprylic acid	7 (10.6)	0.6–39.5
C10:0 (g l^−1^)—capric acid	40.1 (67.3)	3.6–167.3
C12:0 (g l^−1^)—lauric acid	13.3 (21)	0.9–53.8
C13:0 (g l^−1^)—tridecanoic acid	0 (0)	0–0.2
C14:0 (g l^−1^)—myristic acid	1.5 (1.9)	0.1–5.2
C14:1 (g l^−1^)—myristoleic acid	0(0)	0–0.1
C15:0 (g l^−1^)—pentadecyl acid	0.1 (0.1)	0–0.4
C16:0 (g l^−1^)—palmitic acid	2.7 (4.2)	0.4–25.7
C16:1 n-7 (g l^−1^)—palmitoleic acid	0.2 (0.3)	0–2.4
C17:0 (g l^−1^)—heptadecanoic acid	0.1 (0.1)	0–0.1
C18:0 (g l^−1^)—stearic acid	0.4 (0.3)	0.1–6.7
C18:1 *trans* (g l^−1^)—elaidic acid	0 (0)	0–0.3
C18:1 n-9 (g l^−1^)—oleic acid	2.8 (5.0)	0.5-24.8
C18:1 n-7 (g l^−1^)—*cis*-vaccenic acid	0.1 (0.2)	0–1.4
C18:2 n-6 (g l^−1^)—linoleic acid	0.5 (0.7)	0.1–3.4
C18:3 n-3 (g l^−1^)—alpha linoleic acid	0.3 (0.5)	0–2.4
C20:0 (g l^−1^)—arachidic acid	0.2 (0.02)	0–0.5
C20:1 n-9 (g l^−1^)—eicosenoic acid	0 (0.1)	0–0.4
C20:2 n-6 (g l^−1^)—eicosadienoic acid	0 (0)	0–0.3
C20:3 n-6 (g l^−1^)—dihomo-gamma-linolenic acid	0.1 (0.1)	0–0.3
C20:4 n-6 (g l^−1^)—arachidonic acid	0 (0)	0–0.2
C20:3 n-3 (g l^−1^)—eicosatrienoic acid	0.1 (0.1)	0–0.5
C22:0 (g l^−1^)—docosanoic acid	0 (0)	0–0.5
C22:1 (g l^−1^)—erucic acid	0 (0.3)	0.1–0.4
C22:4 n-6 (g l^−1^)—adrenic acid	0 (0)	0–0.6
C24:1 (g l^−1^)—nervonic acid	0 (0)	0–0.6
C22:5 n-3 (g l^−1^)—docosapentaenoic acid	0 (0)	0–0.3
acylcarnitines		
free Carn (µmol l^−1^)	287 (188.6)	3.3–624.2
Carn 2.0 (µmol l^−1^)	43 (83.5)	0.2–198–2
Carn 3.0 (µmol l^−1^)	8.7 (15.7)	0–104.8
Carn 3.0 DC (µmol l^−1^)	0.1 (0)	0–0.1
Carn 4.0 (µmol l^−1^)	4.6 (4.9)	0–23
Carn 4.0 DC (µmol l^−1^)	0 (0)	0–0.3
Carn 4.0 DC3M (µmol l^−1^)	0 (0)	0–0.1
Carn 4.0 iso (µmol l^−1^)	1.3 (1.4)	0.1–27.4
Carn 4.0 OH (µmol l^−1^)	0.6 (0.5)	0–2
Carn 5.0.2 M (µmol l^−1^)	9.6 (12.7)	0.8–153.4
Carn 5.0.3 M (µmol l^−1^)	8.0 (11.1)	0.5–128.5
Carn 5.0 OH (µmol l^−1^)	0.4 (0.4)	0.1–4
Carn 5.1 (µmol l^−1^)	0.6 (0.5)	0.1–4.5
Carn 6.0 (µmol l^−1^)	0.3 (0.3)	0–2
Carn 8.0 (µmol l^−1^)	0.1 (0.1)	0–0.5
phospholipids		
lyso.PC.a.C14.1 (µmol l^−1^)	0 (0.1)	0–0.4
lyso.PC.a.C14.2 (µmol l^−1^)	0 (0)	0–0.1
lyso.PC.a.C16.0 (µmol l^−1^)	0.7 (1.8)	0–4.7
lyso.PC.a.C18.0 (µmol l^−1^)	0.4 (0.7)	0–2.5
lyso.PC.a.C18.1 (µmol l^−1^)	1 (5.4)	0–28.6
lyso.PC.a.C18.2 (µmol l^−1^)	0.1 (0.6)	0–2.9
lyso.PC.a.C18.6 (µmol l^−1^)	0 (0.1)	0–0.9
lyso.PC.a.C20.0 (µmol l^−1^)	0 (0)	0–0.1
lyso.PC.e.C16.3 (µmol l^−1^)	0 (0)	0–0.02
lyso.PC.e.C18.3 (µmol l^−1^)	0 (0)	0–0.3
PC.aa.C16.4 (µmol l^−1^)	0 (0)	0–0.1
PC.aa.C18.6 (µmol l^−1^)	28.5 (32.6)	1.7–98.9
PC.aa.C22.5 (µmol l^−1^)	0.1 (0.1)	0–0.3
PC.aa.C24.0 (µmol l^−1^)	0 (0.1)	0–0.5
PC.aa.C26.0 (µmol l^−1^)	0.3 (1.35)	0–6.6
PC.aa.C26.2 (µmol l^−1^)	0 (0)	0–0.1
PC.aa.C28.3 (µmol l^−1^)	0.1 (0.1)	0–0.9
PC.aa.C30.0 (µmol l^−1^)	5 (8.24)	0.2–31.1
PC.aa.C30.2 (µmol l^−1^)	0 (0)	0–0.04
PC.aa.C32.0 (µmol l^−1^)	8.1 (9.7)	0.3–24.6
PC.aa.C32.1 (µmol l^−1^)	2.7 (8.6)	0–23.8
PC.aa.C32.2 (µmol l^−1^)	0.9 (1.1)	0.1–3.1
PC.aa.C34.0 (µmol l^−1^)	2.9 (2.8)	0.1–8.7
PC.aa.C34.1 (µmol l^−1^)	14.5 (34)	0.4–81.5
PC.aa.C34.2 (µmol l^−1^)	2.1 (5.3)	0–13.1
PC.aa.C34.3 (µmol l^−1^)	0.2 (0.6)	0–1.4
PC.aa.C36.0 (µmol l^−1^)	0.1 (0.2)	0–0.5
PC.aa.C36.1 (µmol l^−1^)	8.9 (19.7)	0.2–54.3
PC.aa.C36.2 (µmol l^−1^)	4.4 (15.1)	0.1–36.7
PC.aa.C36.3 (µmol l^−1^)	1 (4.6)	0–9.4
PC.aa.C36.4 (µmol l^−1^)	0.5 (2)	0–4.8
PC.aa.C38.3 (µmol l^−1^)	4,8 (4.7)	0.6–12.4
PC.aa.C38.4 (µmol l^−1^)	1.1 (1.9)	0–4.7
PC.aa.C38.5 (µmol l^−1^)	0.2 (0.8)	0–2.6
PC.ae.C28.0 (µmol l^−1^)	0.3 (0.8)	0–4.8
PC.ae.C30.0 (µmol l^−1^)	0.3 (0.5)	0–2.6
PC.ae.C30.2 (µmol l^−1^)	0.1 (0.1)	0–0.3
PC.ae.C32.0 (µmol l^−1^)	1.1 (1.2)	0.1–4.1
PC.ae.C32.1 (µmol l^−1^)	0.4 (0.5)	0–2.2
PC.ae.C34.0 (µmol l^−1^)	0.7 (1)	0–2.7
PC.ae.C34.1 (µmol l^−1^)	1.7 (3.3)	0–8.4
PC.ae.C34.2 (µmol l^−1^)	0.7 (1.2)	0–2.9
PC.ae.C36.2 (µmol l^−1^)	1.3 (3.1)	0–7.8
PC.ae.C36.3 (µmol l^−1^)	0.5 (1.3)	0–3.6
SM.a..C20.1 (µmol l^−1^)	0 (0)	0–0.1
SM.a..C20.5 (µmol l^−1^)	0 (0.1)	0–1.1
SM.a..C22.6 (µmol l^−1^)	0 (0)	0–0.1
SM.a..C26.3 (µmol l^−1^)	0 (0)	0–0.4
SM.a..C28.0 (µmol l^−1^)	0 (0.2)	0–1.6
SM.a..C28.1 (µmol l^−1^)	0.5 (2.6)	0–14.9
SM.a..C30.0 (µmol l^−1^)	0.3 (0.7)	0–4
SM.a..C30.1 (µmol l^−1^)	0.7 (1.8)	0–8
SM.a..C31.0 (µmol l^−1^)	0 (0)	0–0.2
SM.a..C32.0 (µmol l^−1^)	0.8 (1.5)	0.1–6.5
SM.a..C32.1 (µmol l^−1^)	1.4 (2)	0–12.7
SM.a..C33.0 (µmol l^−1^)	0.4 (0.6)	0–3
SM.a..C33.1 (µmol l^−1^)	0.3 (0.3)	0–1.2
SM.a.C34.0 (µmol l^−1^)	3 (3)	0.3–12.8
SM.a.C34.1 (µmol l^−1^)	25 (28.5)	3.3–82.6
SM.a.C34.2 (µmol l^−1^)	0.3 (0.4)	0–1.3
SM.a.C35.0 (µmol l^−1^)	0.5 (0.4)	0.1–2
SM.a.C35.1 (µmol l^−1^)	1.4 (1.5)	0.2–5.2
SM.a.C36.0 (µmol l^−1^)	2.7 (2)	0.6–7.2
SM.a.C36.1 (µmol l^−1^)	12.7 (7.7)	2.2–31
SM.a.C36.2 (µmol l^−1^)	0.4 (0.4)	0–1
SM.a.C37.1 (µmol l^−1^)	0.9 (0.6)	0.1–2.1
SM.a.C38.1 (µmol l^−1^)	15.7 (9.3)	3.4–40.1
SM.a.C38.2 (µmol l^−1^)	0.4 (0.4)	0–1.5
SM.a.C39.1 (µmol l^−1^)	1.5 (1.1)	0.3–3.2
SM.a.C40.1 (µmol l^−1^)	5.4 (4.1)	1.2–15.1
SM.a.C40.2 (µmol l^−1^)	1 (1.20)	0.1-3.7
SM.a.C40.3 (µmol l^−1^)	2.1 (2.9)	0.2–10.7
SM.a.C40.4 (µmol l^−1^)	0 (0)	0–0.01
SM.a.C41.1 (µmol l^−1^)	2.4 (2.2)	0.6–7
SM.a.C41.2 (µmol l^−1^)	1 (1.3)	0.1–4.7
SM.a.C42.0 (µmol l^−1^)	0.3 (0.3)	0–1
SM.a.C42.1 (µmol l^−1^)	7.1 (6.9)	1.5–20.8
SM.a.C42.2 (µmol l^−1^)	21.1 (18.9)	3.3–58.4
SM.a.C42.3 (µmol l^−1^)	2.3 (2.7)	0.3–12.1
SM.a.C42.4 (µmol l^−1^)	0.5 (0.7)	0.1–4
SM.a.C43.1 (µmol l^−1^)	0.3 (0.3)	0–0.8
SM.a.C43.2 (µmol l^−1^)	0.7 (1)	0.1–3.9
SM.a.C44.1 (µmol l^−1^)	0 (0)	0–0.1
SM.a.C44.2 (µmol l^−1^)	0.5 (0.6)	0.1–2
organic acids and keto-acids		
lactic acid (µmol l^−1^)	2511 (24596)	123.5–59529.9
pyruvic acid (µmol l^−1^)	745.8 (3066.6)	28–21563.5
succinic acid (µmol l^−1^)	187.6 (454.2)	1.7–2963.6
fumaric acid (µmol l^−1^)	1 (1.2)	0.3–12.6
malic acid (µmol l^−1^)	12.3 (19.6)	1.3–76.6
methyl-malonic acid (µmol l^−1^)	2.9 (5.9)	0.2–21.5
beta-hydroxybutyric acid (µmol l^−1^)	7.2 (10.2)	0.7–100.5
alpha-ketoglutaric acid (µmol l^−1^)	7 (9.3)	1.6–91.7
3-methyl−2-oxobutanoic acid (µmol l^−1^)	4.4 (3.6)	1.1–17.7
3-methyl−2-oxovaleric acid (µmol l^−1^)	1.6 (1.8)	0–50.3
4-methyl−2-oxovaleric acid (µmol l^−1^)	10.9 (22.3)	0–518.3
alpha aminoadipidic acid (µmol l^−1^)	3.3 (2.9)	0.2–17.4
taurine (µmol l^−1^)	55.3 (60.3)	7.7–390.4

**Table 3.** Percentages of individual fatty acids in relation to total fatty acids in milk from *Elephas maximus*.

fatty acid	%
C8:0 (g l^−1^)—caprylic acid	9.7
C10:0 (g l^−1^)—capric acid	55.4
C12:0 (g l^−1^)—lauric acid	18.4
C13:0 (g l^−1^)—tridecanoic acid	0.0
C14:0 (g l^−1^)—myristic acid	2.1
C14:1 (g l^−1^)—myristoleic acid	0.0
C15:0 (g l^−1^)—pentadecyl acid	0.1
C16:0 (g l^−1^)—palmitic acid	3.7
C16:1 n-7 (g l^−1^)—palmitoleic acid	0.3
C17:0 (g l^−1^)—heptadecanoic acid	0.1
C18:0 (g l^−1^)—stearic acid	0.6
C18:1 *trans* (g l^−1^)—elaidic acid	0.0
C18:1 n-9 (g l^−1^)—oleic acid	3.9
C18:1 n-7 (g l^−1^)—*cis*-vaccenic acid	0.1
C18:2 n-6 (g l^−1^)—linoleic acid	0.7
C18:3 n-3 (g l^−1^)—alpha linoleic acid	0.4
C20:0 (g l^−1^)—arachidic acid	0.3
C20:1 n-9 (g l^−1^)—eicosenoic acid	0.0
C20:2 n-6 (g l^−1^)—eicosadienoic acid	0.0
C20:3 n-6 (g l^−1^)—dihomo-gamma-linolenic acid	0.1
C20:4 n-6 (g l^−1^)—arachidonic acid	0.0
C20:3 n-3 (g l^−1^)—eicosatrienoic acid	0.1
C22:0 (g l^−1^)—docosanoic acid	0.0
C22:1 (g l^−1^)—erucic acid	0.0
C22:4 n-6 (g l^−1^)—adrenic acid	0.0
C24:1 (g l^−1^)—nervonic acid	0.0
C22:5 n-3 (g l^−1^) docosapentaenoic acid	0.0

### 3.2. Associations with lactation

**Figure 3.**
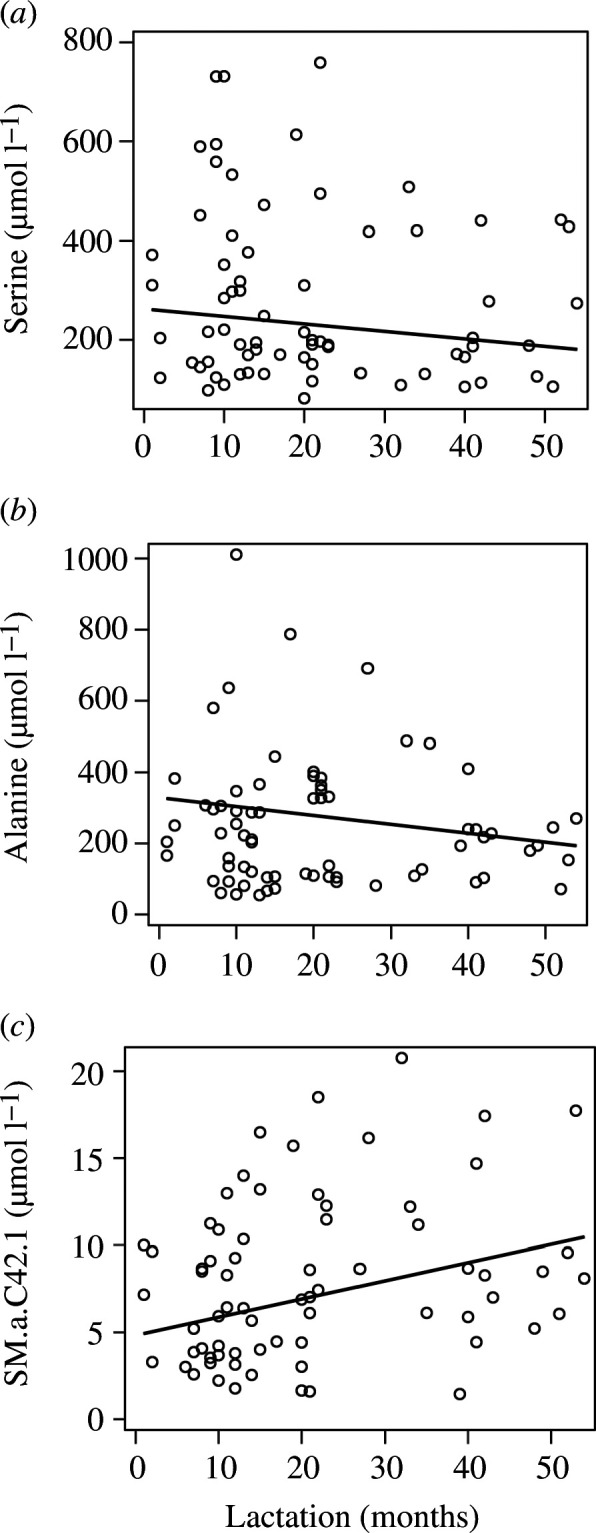
Statistically significant linear regressions between the concentration of the most abundant milk metabolites (serine (*a*), alanine (*b*) and SM.a.C.42.1(*c*)) and the lactation time (months postpartum). Individual circles represent raw values. Solid lines represent the predicted values according to each fitted model. Each panel represents adjusted repeated measure models including the outcome variable (milk metabolite), the lactation time, parity and sex of the calf (species: *Elephas maximus*).

Associations with the lactation time (month postpartum) were tested individually for all the most abundant compounds (alanine, aspartic acid, glutamate, glycine, proline, serine, capric acid, lauric acid, oleic acid, carnitine, acetyl-carnitine, PCaaC18.6, SM.a.C42.1, SM.a.C42.2, SM.a.C38.1, SM.a.C36.1, lactic acid, pyruvic acid, succinic acid and taurine), total fatty acids and total proteinx. The majority of these compounds did not show significant association with the lactation period: total protein and total fatty acids (electronic supplementary material, figure S1), glutamate, glycine, aspartic acid, carnitine, SM.a.C38.1, lactic acid, pyruvic acid and succinic acid; while others showed changes as lactation progressed. In this context, we found significant associations with lactation time for alanine (*p* = 0.009, *β* = −0.004), serine (*p* = 0.038, *β* = −0.004), lauric acids (*p* = 0.017, *β* = 0.007), PCaaC18.6 (*p* = 0.034, *β* = −0.005), SM.a.C42.1 (*p* = 0.001, *β* = 0.006) and SM.a.C42.2 (*p* = 0.001, *β* = 0.006). However, when adjusting the models as described in the methods section, we found that only SM.a.C42.1 (*p* = 0.004, β = 0.006), alanine (*p* = 0.037, *β* = −0.004) and serine (*p* = 0.040, *β* = −0.004) were significantly associated with the lactation time. Oleic acids showed a positive trend (*p* = 0.082). Adjusted significant associations with the lactation time are shown in figure 3. When exploring associations between each metabolite class and the lactation time through multivariate (MANOVA) analysis we found different results between models that included elephants 6 and 17 (that gave birth to a new calf during milk collection), vs models that did not include them. Models that included early lactation samples of these two females showed overall significant associations between the lactation time and amino acids (Wilks’ Lambda *p* = 0.040) and a trend for the TCA compounds (Wilks’ Lambda *p* = 0.079) when unadjusted, while they showed significant associations between lactation time and fatty acids (Wilks’ Lambda *p* = 0.040) when adjusted for sex of the calf and parity. Multivariate models that did not include samples from elephants 6 to 17 showed no significant association between the lactation time and any of the metabolite classes when unadjusted, but became significant for TCA compounds (Wilks’ Lambda *p* = 0.034) and showed a trend for the carnitines (Wilks’ Lambda *p* = 0.089) when adjusted for sex and parity. Nonetheless, for the classes that showed overall significant associations with the lactation time, no significant differences were found for any specific compound over lactation.

### 3.3 Associations with maternal-calf characteristics and collection time

The adjusted mixed models for the most abundant compounds showed significant positive associations between parity and total protein (*p* = 0.001, *β* = 0.192), SM.a.C42.2 (*p* = 0.018, *β* = 0.029), and acetyl-carnitine (*p* = 0.003, *β* = 0.076). Additionally, carnitine was positively associated with male sex of the calf (*p* = 0.015, *β* = 0.121). Multivariate adjusted models (including early samples from elephant 6 to 17) showed an overall trend between fatty acids and parity (*p* = 0.050) and calf sex (*p* = 0.060). Multivariate adjusted models not including samples from elephant 6 to 17 showed an overall significant trend between carnitines and calf sex (*p* = 0.062).

## 4. Discussion

With the present study, we aimed at characterizing the nutritional and metabolite composition of Asian elephant milk collected from animals experiencing their natural habitat and diet. To the best of our knowledge, this study is the first using extensive metabolomic analysis to characterize milk from Asian elephants. The study was able to identify the presence and concentration of 160 metabolites (22 amino acids, 27 fatty acids, 15 acyl-carnitines, 84 phospholipids and 12 compounds of the TCA cycle). The study showed that some metabolites in each category were particularly abundant compared to others, suggesting a possible role in the healthy development of Asian elephant calves. Some of the compounds tested showed an association with the lactation time, suggesting that time of exposure to specific milk components might also be crucial to ensure normal growth and development of the offspring and/or signalling a shift in the metabolism of the mammary gland. Finally, we also observed that a few components were significantly associated with the parity and sex of the offspring, suggesting that both these factors have an effect on lactation physiology and should be taken into account when analysing milk composition.

To the best of our knowledge, this was the first study yielding a comprehensive profiling of the Asian elephant milk metabolome. Our group previously analysed milk from six females belonging to the same population of Asian elephants (i.e. MTE owned semi-captive animals), finding that fat, protein, ash and vitamin E content of the milk varied with the lactation month and season, possibly in relation to the variation of nutrients in the forage consumed by mothers [17]. This report was the first on milk composition of Asian elephants living in their natural habitat. With the present study, we sought to analyse a substantially bigger sample number and to identify new components present in the Asian elephant milk that might have a crucial role in calf growth and development. In fact, previous studies on this species only analysed single classes of components including glucosamine [28], oligosaccharides [41,42,63], or generic nutritional composition [17,44]. These studies were carried out on a limited number of animals (between 1 and 6 animals) and all, except our previous study, collected milk from animals held in captive environments. More research has been conducted on African elephant (*Loxodonta africana*) milk [16,43,52,53,64,65], including a metabolomic analysis, which was recently published [65]. Nonetheless, this study was also carried out on a limited number of animals (*n* = 3), and while the animals were living in their natural habitat, the study only quantified 36 metabolites, 14 of which remained unidentified. The present study is therefore, to date, the most comprehensive metabolomic analysis of elephant milk.

Our study found that Asian elephant milk was very rich in glutamate and glycine. These two amino acids were over 10 times more concentrated than the second most abundant amino acids (e.g. alanine) and over 300 times more concentrated than the least abundant amino acids (e.g. methionine) in the analysed milk samples. Glutamate is also the most abundant free amino acid found in milk from humans and other animals [66,67] and recent research on African elephant milk also confirms high concentration in this taxa [65]. In this publication the authors suggest that the high concentration of glutamate might be linked to the gluconeogenic nature of this metabolite and linked to the high saccharide content in the milk, although we cannot confirm or disprove this. In relation to our results, the high concentrations of glutamate in comparison to other amino acids might be explained by the mammary gland physiology during lactation (although no data is available for this species), which entails a substantial catabolism of branched-chained amino acids (leucine, isoleucine, valine) and arginine for the production of glutamate [68–70]. As previous literature describes, dietary glutamate is used by the newborn intestinal mucosa as a source of energy, glutathione and other essential amino acids [66,68]. Similarly, glycine is also abundant in milk from other species (e.g. pigs) [70] and a substrate for the synthesis of glutathione, protein and nucleic acids [71,72]. Furthermore, it is interesting to note that both glutamate and glycine are molecules with a neurotransmitter function [73–75] and although research on whether dietary glycine has any neuromodulatory function seems to be particularly scarce, research has shown that dietary glutamate can have a positive influence on brain development [76].

In relation to the lipid profile, our samples contained around 7% of total fatty acids. This was lower compared to the total fat% previously reported by our group (15%) [17]. The present study only quantified fatty acids, which inevitably led to a slight underestimation of the milk total fat content. Moreover, the total fat% reported by various publications seems to be generally variable [53] and our previous study found that the fat component was indeed one of the most affected by the dietary intake of the mother [17]. The fact that only 20 ml of milk was collected from mothers could also affect the total fat concentration in the sample. Indeed, it is known that fore milk (milk at the beginning of the feed) and hindmilk (milk at the end of the feed) significantly differ in terms of fat content [77,78]. Fat content in elephant milk is much higher than that found in milk from humans and some farmed species (e.g. cow, horse, goat), while it is closer to the milk from buffalos (5–15%) and sheep (4.9–7%) [79]. In line with what has been reported by previous literature on elephant milk [16,64,65] saturated fats made up the majority of the fatty acid profile in our samples, with capric acid being the most abundant and making up almost 60% of the total fatty acid content. Once again, it is interesting to note that capric acid is a neuroactive component that can serve as alternate source of energy to the brain thanks to its ability to cross the blood-brain barrier [80], suggesting that Asian elephant calves, or all elephant calves, display increased needs for this fatty acid. Additionally, capric acid also seems to bring benefits to intestinal health [81], which is often a problem in hand-reared Asian elephants [82]. Yet, milk replacers currently available on the market for the hand-rearing of Asian elephants contain very variable amounts of this compound and an unknown amount of glutamate or glycine [31], which the present study also found in high concentration in elephant milk.

Our study also identified a number of short- and medium-chain acyl-carnitines (C2-8) in the milk of Asian elephants. Acyl-carnitines are fatty acid metabolites involved in energy production and metabolic pathways [83]. Our milk samples displayed high concentrations of carnitine and acetyl-carnitine (Carn.2). Few studies in the literature describe the acylcarnitine profile of milk from different animals and of different formulas [84,85]. These limited data suggest that carnitine profile can change significantly across species. For instance, milk from water buffalo (*Bubalus bubalis*) was reported to contain much higher concentrations of acyl carnitines (and similar to concentrations observed in our samples) compared to milk from cows [84]. As carnitines play an important role in energy production, especially during early life [83], when newborns are less efficient in endogenously synthesizing them [83,86,87], such variability could have important implications for the health outcomes of hand-reared animals. Previous research in rats suggests that mothers deplete their body reserves of carnitines, especially during early lactation to transfer them to the newborn through the milk [88]. While in our study we did not find an association between acylcarnitines and the lactation period, our population was predominantly constituted by mothers in mid-late lactation (as discussed more extensively later in the discussion).

The analysis of the phospholipid metabolites in our samples showed the presence of phosphatidylcholines (PC 96 µmol l^−1^) and sphingomyelins (SM 113.6 µmol l^−1^). These are commonly known to be among the abundant species of phospholipids in milk, and research on human milk carried out already in the 1980s showed similar concentration to those we identified in the Asian elephant [89]. Nonetheless, more recent research shows that the phospholipid profile can be different across mothers of the same species [90–92] and across mothers of different species [93]. Particularly human milk seems to have higher amounts of both PC and SM compared to farm animals [93]. The similarities between human and elephant milk in this context may once again suggest a role of these compounds in the neurodevelopment of elephants’ large brain and cognition. Phospholipids are the major constituents of cell membrane, and the literature indeed suggests that dietary phospholipids in early life can affect cognition and brain development in both humans [11] and other mammals with complex brain structure such as pigs [94]. In the mammary gland, phospholipids are essential for the secretion of lipids, carrying out important structural functions as part of milk fat globules [92,95]. Finally, milk phospholipids are known to act as an emulsifier for liposoluble substances affecting their digestibility [92,96]. This is particularly important to note in relation to the digestibility of milk replacers for orphaned calves where the phospholipid composition might not replicate that of maternal milk.

Our study found that Asian elephant milk contained high amounts of lactate, pyruvate and succinate. These three organic acids are part of the glucose metabolism and as such cover important roles in ATP production and energy metabolism [97–99]. While these metabolites are mostly used as biomarkers for various diseases [86], previous research on dairy cows suggests that the milk metabolome is a good proxy for the mammary gland metabolome and can provide information on the mammary gland metabolic status during lactation [100]. Furthermore, the same study showed that during lactation, gluconeogenesis, pyruvate metabolism, the TCA cycle and the aspartate metabolism pathways were most active in the mammary gland [100]. These findings are in line with some of the compounds that we saw as most abundant in our milk samples, including lactate, pyruvate, carnitine, glutamate and glycine. Oral administration of these organic acids of the TCA cycle can play an important role in gut physiology, including food intake regulation [101], improved mitochondrial activity in skeletal muscle [102] and glucose homeostasis [103] in the newborn. Nonetheless, such high lactate and pyruvate concentrations in our samples could also be owing to the activity of lactic acid bacteria after sample collection. In fact, despite the efforts made to preserve sample quality, the collection of samples from Asian elephants in a low-income country presented its challenges and we cannot exclude bacterial proliferation and contamination in our samples. Taurine was also present in our samples in relatively high quantities compared to other metabolites, although in much lower concentrations compared to what has been reported in human milk [104]. The role of taurine in the perinatal period is well established [10,22,23]. Thus, its presence in milk of the Asian elephants was expected. Previous literature on rats suggests that taurine present in maternal milk is both transported from the blood stream and synthesized in the mammary gland from methionine, cysteine (both of which we found in small amounts in our milk samples) and homocysteine [105]. Considering the implications for taurine in the developmental origin of health and diseases in humans and other animals [105], particularly from a behavioural and neurological standpoint, it seems logical to think that this would be the same for Asian elephant calves. This observation could be particularly relevant for captive and semi-captive restrained animals where environmental stress might cause taurine depletion in mothers, as previously observed in mice [106], or where the provision of inadequate early life nutrition in the absence of maternal milk might impair calf development, as previously shown in monkeys [107].

Lastly, our results suggest that the majority of the tested compounds remained stable during lactation, while only three compounds showed linear associations with the lactation stage (alanine, serine and SM.a.C42.1) when the analysis was adjusted for factors affecting lactation, such as parity and sex of the calf. While unadjusted models found a significant linear association with various compounds (alanine, serine, lauric acid, SM.a.C42.1, PC.aa.C18.6, SM.a.C42.2) these associations might not be reflecting true changes in the milk composition as they fail to take into account major factors that impact on the variability of milk composition across mothers [13,46,47,108,109]. Furthermore, multivariate analysis also showed that the association between milk composition and the lactation stage were mild and mostly non-significant. Our results are not fully aligned with previous studies where a linear increase of lipid and protein content in the milk across lactation had been suggested, while we can confirm that no clear pattern was reported with the phospholipids as previously reported by Kobeni *et al*. [16,17,44,53]. Overall, this discrepancy might suggest that the diversity and limitations that affect this and previous studies in the field (e.g. the use of few animals and the pooling of lactations to describe the variation of milk components over time) need to be addressed in order to obtain a full characterization of elephant milk composition across the lactation time. In light of this, we strongly advise that future research focuses on conducting more thorough research on how the lactation stage might affect milk composition in the Asian elephant through a longitudinal study design including multiple mothers followed-up from birth to late lactation. Additionally, the study should include taking into account maternal and offspring factors that have been demonstrated or suggested to affect the physiology of the mammary gland during lactation. For instance, parity is an essential factor to take into account in this context as different parity corresponds to different degrees of mammary gland maturation [108] which reflects in milk yield and composition. The sex of the offspring is also emerging as an important factor in terms of maternal investment related to lactation, as shown by studies on various species [13,46,47,109]. As a matter of fact, significant associations with both parity (total protein and acetyl-carnitine) and the sex of the offspring (carnitine) were evidenced by our analysis. In this context the sex-specific difference in carnitine, showing increased carnitine levels in milk produced for male calves might suggest either or both an increased maternal metabolism/investment when feeding male calves, or increased male offspring needs, possibly driven by increased energy expenditure compared to female calves. A deeper exploration of these factors is warranted to understand the origins of milk compositional variations across and within mothers.

Strengths of the present study include the novelty of the analysis carried out, which resulted in the identification and quantification of compounds in the milk of Asian elephants that were never identified and quantified before. In fact, to our knowledge, the study constitutes the most comprehensive metabolite profiling of Asian elephant milk composition to date. The semi-captive environment in which the study animals live and the sample size are further strengths of the study, given the fact that most of the previous research on the Asian elephant focused on a few captive animals. On the other side, the possibility of contamination from environmental bacteria and the scarcity of milk collected during early lactation are weak points of our research design. The secluded location of the timber camps and poorly developed transport networks presented challenges for the collection of samples, but all efforts were made to minimize sample spoilage and contamination. Instructions for sample collection were provided to local staff, and refrigeration was arranged during transport from the collection site to the research office, where the samples were stored until shipping to Finland.

## 5. Conclusion

The present study sheds light on the presence and quantity of metabolites in the milk of Asian elephants living in their natural habitat. The study found that there are compounds such as glutamate, glycine, capric acid, phosphatidylcholine, sphingomyelin, carnitine, lactate, pyruvate and succinate that are present in rather high concentrations in the milk from this species. Such high concentrations might be linked to specific physiological roles that these compounds cover during early life and that might be crucial for the survival of Asian elephant calves. Additionally, our study found associations between few of these compounds and the lactation stage, although milk composition mostly seemed stable over the lactation period considered in our sample. Associations found between milk composition and factors such as parity and sex of the offspring confirm the involvement of these factors in the physiology of the lactating mammary gland and need to be further explored. Given that our study highlighted the presence and composition of abundant compounds in the milk of this species, future research should investigate whether currently marketed milk replacers contain similar quantities of these compounds and if not, whether these can be safely and timely introduced for the successful hand-rearing of Asian elephant calves with no access to maternal milk. Finally, further research should investigate whether and how these components vary across different mothers and whether variability in their concentration is linked to maternal and calf health.

## Data Availability

Owing to proprietary reasons and in line with agreements between the University of Turku and Myanma Timber Enterprise (MTE) the authors are not able to publish the dataset used for the study. Access to the data can be requested to the data handlers for the study (Prof Virpi Lummaa: virpi.lummaa@utu.fi, and Dr. Mirkka Lahdenperä: mirkka.lahdenpera@utu.fi). Supplementary material is available online [110].
